# Residential Pesticide Usage in Older Adults Residing in Central California

**DOI:** 10.3390/ijerph8083114

**Published:** 2011-07-25

**Authors:** Mary N. Armes, Zeyan Liew, Anthony Wang, Xiangmei Wu, Deborah H. Bennett, Irva Hertz-Picciotto, Beate Ritz

**Affiliations:** 1 Department of Epidemiology, School of Public Health, University of California, Los Angeles, P.O. Box 951772, Los Angeles, CA 90095, USA; E-Mails: mnarmes@ucla.edu (M.N.A.); zeyanliew@gmail.com (Z.L.); anthony.epi@gmail.com (A.W.); 2 Department of Public Health Sciences, University of California, One Shields Avenue, TB 169, Davis, CA 95616, USA; E-Mails: xmwu@phs.ucdavis.edu (X.W.); dhbennett@ucdavis.edu (D.H.B.); ihp@phs.ucdavis.edu (I.H.-P.)

**Keywords:** pesticides, residential exposure, exposure-related behavior, lifetime use, older adults

## Abstract

Information on residential pesticide usage and behaviors that may influence pesticide exposure was collected in three population-based studies of older adults residing in the three Central California counties of Fresno, Kern, and Tulare. We present data from participants in the Study of Use of Products and Exposure Related Behaviors (SUPERB) study (N = 153) and from community controls ascertained in two Parkinson’s disease studies, the Parkinson’s Environment and Gene (PEG) study (N = 359) and The Center for Gene-Environment Studies in Parkinson’s Disease (CGEP; N = 297). All participants were interviewed by telephone to obtain information on recent and lifetime indoor and outdoor residential pesticide use. Interviews ascertained type of product used, frequency of use, and behaviors that may influence exposure to pesticides during and after application. Well over half of all participants reported ever using indoor and outdoor pesticides; yet frequency of pesticide use was relatively low, and appeared to increase slightly with age. Few participants engaged in behaviors to protect themselves or family members and limit exposure to pesticides during and after treatment, such as ventilating and cleaning treated areas, or using protective equipment during application. Our findings on frequency of use over lifetime and exposure related behaviors will inform future efforts to develop population pesticide exposure models and risk assessment.

## 1. Introduction

Pesticide exposure has been associated with increased risk of adult cancers [1], endocrine disruption [2,3], and neurological disorders such as Parkinson’s disease [4,5]. Two studies using urine samples from the 1999–2000 National Health and Nutrition Examination Survey (NHANES) reported that up to 76% and 96% of the samples tested positive for metabolites of pyrethroids and organophosphates, both chemicals commonly found as ingredients in residential and agricultural pesticide formulations [6,7]. It was reported that 102 million pounds of pesticide active ingredients were applied in homes and gardens in the United States in 2001 [8]. National and regional studies with self reports and/or environmental samples found that a majority of US households used pesticides in their homes, yards, and/or gardens during or in the year prior to data collection [9–13]. This widespread residential pesticide use suggests that a significant portion of the population may be exposed to pesticides in their homes. However these studies did not report application patterns or information about longer term and lifetime use. Residential pesticide use data that includes information about application methods and patterns, total lifetime use, and other exposure related behaviors are needed for risk assessment and for developing population exposure models.

In recent years several models have been developed to estimate residential exposure to pesticides [14]. One model developed by the US Environmental Protection Agency (EPA) is the Stochastic Human Exposure and Dose Simulation (SHEDS), which uses factors such as frequency of application, application type, and co-occurrence of application types to predict exposures for specified scenarios [15]. However these models omit several factors that may affect exposure estimation such as patterns of lifetime pesticide usage, areas of a home being treated, location for pesticide storage in a home, protective measures used during application, ventilation during and after and cleaning after treatment. Our study provides information on many of these omitted factors.

We recently reported on pesticide application methods and behaviors in households with young children [16]. Here we instead focus on current and lifetime residential pesticide use in older adults, an age group that may also be especially vulnerable to toxins, such as the nervous system’s greater sensitivity to neurotoxins [17], and other age-related factors.

To gain a better understanding of patterns and methods of residential pesticide use in older adults we will utilize information from three different studies, the Southern California cohort of elderly from the U.S. EPA funded Study of Use of Products and Exposure Related Behaviors (SUPERB) [18] and the population control subjects interviewed for the Parkinson’s Environment and Genes (PEG) and The Center for Gene-Environment Studies in Parkinson’s Disease (CGEP) studies. We have focused specifically on a population of older adults residing in an area of intense agricultural activity; therefore this population may also be exposed to pesticides from agricultural and occupational sources, as well as from residential pesticide use. For the purposes of this paper we use the term ‘pesticides’ for any chemical used to eliminate and/or control plant, animal, or insect pests in and around the residence. We hope that this descriptive study of residential pesticide use and exposure related behaviors will inform future studies of cumulative pesticide exposure to pesticides from multiple sources, as well as inform risk assessment and future modeling of pesticide exposure. We will describe (1) the prevalence and frequency of current and lifetime use of residential pesticides; (2) how pesticides were applied; and (3) pesticide application related behaviors that may affect exposure.

## 2. Methods

### 2.1. Study Cohort

**Figure 1.**
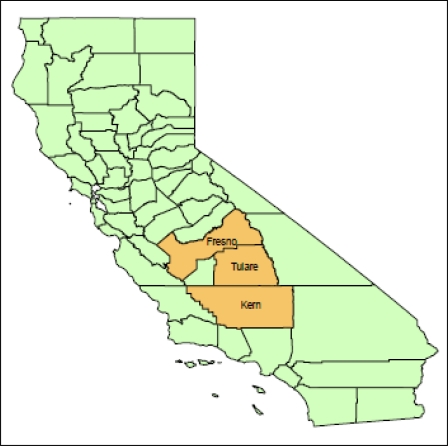
Map of the State of California highlighting Fresno, Kern, and Tulare Counties, the three counties from which PEG, SUPERB, and CGEP recruited participants.

All three studies that contributed data are based on surveys of older adults residing in Fresno, Kern, and Tulare counties, located in California’s Central Valley, an area of intense agricultural activity (Figure 1). All three studies recruited participants from all three counties specifically selected as study areas because they are similar both demographically and in terms of intensity of agricultural activity. Since these studies collected slightly different information on residential pesticide use, we present data from all three studies in order to obtain a more comprehensive picture of lifetime pesticide use and behaviors related to pesticide use.

#### 2.1.1. SUPERB Recruitment

The SUPERB study population consists of residents, age 55 years or older, recruited in three rounds from the three target counties in the California Central Valley. In the first round, beginning in November 2006, we recruited 55 participants by phone and 65 by mail; in round two, 47 participants were enrolled using a mailed screening questionnaire and follow-up phone calls. In the last round of recruitment, 306 door-to-door solicitations were conducted and enrolled 18 participants. In total, 159 participants were enrolled and 154 completed the baseline interview on pesticide use, 153 participants were used for our analysis. A more detailed description of the SUPERB study methods is available elsewhere [18].

#### 2.1.2. PEG Recruitment

Eligible population controls for the PEG study were at least 35 years of age, residents of Fresno, Kern, or Tulare counties, had lived in California for at least five years prior to the study, and did not have Parkinson’s Disease. Initially in 2001, for the PEG study (2001–2007) our population controls age 65 or older were randomly selected from Medicare lists for the three counties and younger subjects from tax assessor parcel listings. However, the passage of the Health Insurance Portability and Accountability Act (HIPAA) prohibited the use of Medicare data for these purposes; therefore we limited our recruitment strategy to using tax assessor parcels only, for subsequent enrollment. Residential parcels were randomly selected and names and phone numbers were obtained from Internet searches and marketing companies. Potential participants were contacted by phone or mail and screened for eligibility by trained study staff. Only one member of each household selected was eligible to enroll. Overall 1,038 potential participants were contacted by mail and/or telephone, after screening 817 were eligible, and 403 were enrolled and completed the interview on residential pesticides. After limiting to ages 50 and older, 359 PEG participants qualified for this analysis.

#### 2.1.3. CGEP Recruitment

Eligibility criteria for control recruitment in the CGEP study (2008–2011) were the same as for PEG and we again relied on tax assessor’s parcel listings to randomly select residences. But for CGEP, population control subjects were recruited through home visits made by trained field staff, who determined eligibility and enrolled the controls at the door step. This was done in an effort to increase enrollment success and representativeness of the sample population compared to the general population in the three target counties. Recruitment of CGEP participants is ongoing, as of January 2011: 6891 homes were visited, 1355 individuals were found possibly eligible and 601 enrolled. At the time of analysis 314 interviews with data on home pesticide use were available for our analysis. Limiting to individuals age 50 and older, the sample used for our analysis contained 297 CGEP participants.

### 2.2. Data Collection

All studies (SUPERB, PEG, and CGEP) used telephone interviews to obtain data on pesticide exposure. Interviews for PEG and CGEP were conducted by trained staff at the University of California, Los Angeles. PEG interviews were conducted from November 2001 to November 2007 and CGEP interviews used for this analysis were conducted from March 2009 to December 2010. Interviews for SUPERB were conducted by trained staff at the University of California, Davis. The SUPERB study collected data in three tiers, but only data from the telephone interviews administered in the first year of Tier 1 will be used here. SUPERB Tier 1, year 1 interviews were conducted from October 2006 to May 2008.

In all three studies we recorded product names, purposes of use, and frequency of indoor and outdoor pesticide use, professional pesticide applications, and applications of pet flea/tick treatments. The questionnaires used for all three studies asked comparable, if not the same questions for each of the above items of interest (Table 1).

Only SUPERB collected information on the area and rooms treated, the size of treated indoor and outdoor areas, and information on cleaning after and ventilation practices during and after indoor applications. Prior to the SUPERB interview, participants were given a list with pictures of current pesticide products in order to facilitate the recall of product names and brands. Most data collected for SUPERB pertained specifically to insecticide usage in the last year. SUPERB also contained a small subset of questions pertaining to indoor and outdoor pesticide use frequency from ages 18–50, for this age period it was possible for participants to report use of any type of pesticide. PEG and SUPERB recorded self-reported information on pesticide storage and personal protection methods used during application.

The emphasis of PEG and CGEP was less on recent but more on lifetime residential pesticide usage. Thus, product names, purposes of use, and frequency of use of specific pesticides (both indoor and outdoor) were collected for four periods of the participant’s lifetime: young adult (16–24 years of age), adult (ages 25–44), middle age (ages 45–64), and senior (age 65 and older). It is important to note that not all participants had reached the age of 65 at the time of interview, therefore questions regarding the 65 and older period often had a smaller sample size than the three younger age periods.

PEG and CGEP participants did not receive additional materials to aid with their recall of product names and brands. We relied only on the product names recalled by participants and the purpose of using the reported pesticide. If a participant could not remember data was recorded as missing. Relying on pesticide product information and years of use reported by PEG/CGEP participants, we utilized the California Department of Pesticide Registry (CDPR) online database to identify the pesticide products’ active ingredients [19]. The CDPR database contains information on pesticide formulations sold in California as far back as 1945. This extensive database allowed us to identify active ingredients for pesticides that participants reported using throughout their lifetime in a time specific manner. When participants did not provide sufficient information to accurately identify the correct product, active ingredient information was treated as missing. When the product was identifiable, among active ingredients, the one with the highest concentration was considered the main active ingredient. A chemical class was then assigned to the corresponding main active ingredient of a particular product. Chemical class information was primarily identified from CDPR and the Pesticide Action Network (PAN) Pesticide Database [19,20]. For analysis, we assigned active ingredients to one of the following chemical classes: pyrethroids, organophosphates, nitrogen containing lactones, carbamate, halogenated, metals/inorganic compounds, organochlorine, and botanicals (including pyrethrum). In some cases a product’s chemical composition changed over time, thus we assumed subjects were exposed to all possible active ingredients in a product during the reported years of usage. Depending on the composition of the product, some were assigned more than one main ingredient and chemical class.

### 2.3. Data Analysis

We used data from all three studies to evaluate pesticide use throughout a person’s lifetime. We generated frequencies and percentages to describe the prevalence of various pesticide usage and exposure related behaviors. Most variables were multinomial rather than normally distributed. We also compared pesticide use within age groups by education and race. In many instances breaking our study population into smaller subgroups for comparison purposes created small cell sizes, necessitating the use of Fisher’s exact test. Another goal was to compare pesticide use during younger and older adulthood. In order to pool data from all three studies, it was necessary to reorganize the data, defining younger adulthood and older adulthood in each study in a slightly different manner prior to pooling; *i.e*., as <45 *vs*. ≥45 for CGEP and PEG and <50 and ≥50 years of age in SUPERB. Because our data on use of pesticides during younger and older adulthood was dichotomous (yes/no) we employed the Phi coefficient to compare pesticide usage across age groups. Data analysis was conducted using SAS 9.2 (SAS Institute Inc., Cary, NC, USA).

## 3. Results and Discussion

### 3.1. Demographics

The SUPERB and PEG subjects recruited by mail and phone were much less diverse than the CGEP population and the general population of the three counties. SUPERB and PEG included at least 80% whites and 9–11% Hispanics while the door-to-door recruitment in CGEP enrolled 59% white and 25% Latino participants. The population of Fresno, Kern, and Tulare, according to Census 2000 data is comprised of 68% (non-Hispanic) whites and 21% Hispanics/Latinos [21]. All other races contributed a very small percentage to the study populations (less than 5–10%). Both SUPERB and CGEP recruited a greater proportion of female participants than PEG. Only PEG’s gender distribution was similar to the Census 2000 data from the three counties (54% female) [21]. While the majority of participants had at minimum received a high school diploma, and PEG and CGEP were able to recruit more subjects with ≤12 years of education, all three populations slightly over-enrolled subjects with more than 12 years of education compared to the general population according to the 2000 census data. Only 45% of residents above age 45 in the three counties had more than 12 years of education, while in our three study populations 51–62% of participants reported more than 12 years of education [21]. More than 50% of PEG and CGEP participants were 60–79 years of age (PEG: mean 69.2 SD ±9.8, CGEP: mean 67.4 SD ±9.4), compared to SUPERB, in which more than 50% of participants were 50–69 years of age (mean 65.6 SD ±8.5) (Table 2).

### 3.2. Lifetime Pesticide Use

Overall 89% of participants from all three study populations used indoor and/or outdoor pesticides at some point during their lifetimes, 72% of participants from all three studies reported ever using pesticides outdoors and 74% ever using pesticides indoors at any point during their lifetime. Our data on ever use frequency are comparable to previous studies that investigated residential pesticide use. In an older study by Savage *et al*. (1981) for the EPA region IX, which contains California and other western states, 62% of all participants reported ever using pesticides in the yard, 28% reported using pesticides in the garden, and 83% of households reported ever using pesticides inside their home within 12 months of being interviewed [11]. A more recent study conducted by Colt *et al*. (2004) in Los Angeles, Detroit, Iowa, and Seattle reported that 94% of subjects had ever used insecticides in or around their current or former residences during a 30 year period prior to interview [9]. In our study we found that 18% of individuals had used indoor pesticides only during their lifetime, 16% had used outdoor pesticides only, and 56% had used both. We speculate that many factors may contribute to this pattern of use, such as type of dwelling and urban versus suburban or rural address. Unfortunately our three studies did not collect any or comparable information about such factors.

We also examined frequency of pesticide use by race and education in each of the four age periods, young adult (16–24), adult (25–44), middle age (45–64), and senior (≥65). We found that there was no statistically significant difference of outdoor pesticide frequency of use by race. For indoor pesticide frequency of use, the only statistically significant finding (Fisher exact N = 388, p= 0.03) was that non-whites, ages 16–24, appeared to use indoor pesticides more frequently than whites ages 16–24. When examining frequency of outdoor pesticide use by education the only statistically significant difference in use during lifetime was seen after age 65 (Fisher exact N = 285, p = 0.003), such that individuals with <12 years of education appeared to use outdoor pesticides more frequently. Individuals with <12 years of education used indoor pesticides significantly more frequently throughout most of their adult lives (Fisher exact adults: N = 472 p < 0.001; mature adults: N = 475 p = 0.004; seniors: N = 282, p = 0.02)

**Figure 2.**
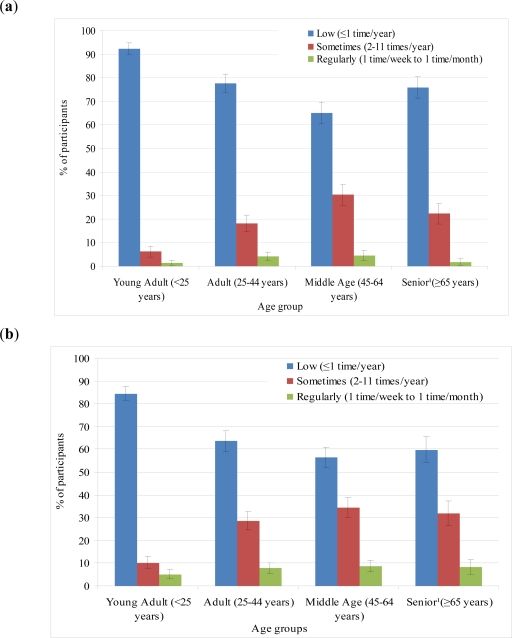
(**a**) Frequency of outdoor pesticide use over lifetime, among PEG and CGEP participants among those who ever reported using outdoor pesticides (N = 439 *); (**b**) Frequency of indoor pesticide use over lifetime, among PEG and CGEP participants among those who ever reported using indoor pesticides (N = 476 **). * Outdoor: Not all participants responded for each age group Young adult N = 434, Adult = 433, Middle Age N = 436, Senior N = 340); ** Indoor: Not all participants responded for each age group Young Adult N = 469, Adult N = 472, Middle Age N = 475, Senior N = 282); ^1^ Not all participants had reached the age of 65 at the time of interview.

In PEG and CGEP, lifetime frequency of pesticide use, both indoors and outdoors, increased with increasing age (Figure 2). Frequency of pesticide use is greatest during middle age (ages 45–64). We speculate that increased use of pesticides during middle age may be a reflection of changes in lifestyle during middle age. For example, individuals in middle age may be more likely to own their homes, whereas young adults (16–24) may still live with family or rent an apartment. A homeowner may be more likely to apply residential pesticides in his/her home compared to an apartment tenant who does not have or take care of yards and gardens and may rely on a landlord to eliminate pests.

Relying on information from all three studies we found that 50% of participants had ever used outdoor pesticides during younger adulthood (16–44 years) and 63% had ever used outdoor pesticides during older adulthood (≥45 years). More than half of all participants had ever applied pesticides indoors during both younger and older adulthood (56% and 61% respectively). Outdoor and indoor pesticide use during younger adulthood (yes/no) was positively correlated with pesticide use (yes/no) during older adulthood (r_φ_ = 0.41, p < 0.0001 and r_φ_ = 0.37, p < 0.0001; respectively) (Table 3).

Data on pesticide use in younger and older adulthood were collected differently in PEG and CGEP versus SUPERB. In SUPERB use in older adulthood was evaluated only as use in the past year, whereas in PEG and CGEP use was evaluated during ages ≥45 years. Thus, the correlation between use during younger and older adulthood in SUPERB was far smaller than in PEG and CGEP. Therefore the correlation using data from all three studies combined, may be weaker than if data on pesticide usage had covered a longer period of older adulthood in SUPERB .

Our findings suggest that people are likely to use pesticides throughout their lifetimes, albeit at relatively low frequency. While the overall frequency of pesticide use was low, as people age there seems to be an increase in use. Our results also show that use of pesticides at younger ages may be related to use of pesticides at older ages. However, further studies are necessary to examine whether general attitudes toward pesticide use, which may be influenced by occupation or education, influence an individual to adopt and continue residential pesticide use throughout lifetime. Additionally it appears that at certain ages, race and education may also influence frequency of pesticide use. Higher use frequencies among older adults are important since pesticides may act more strongly as neurotoxins due to the nervous system’s inability to properly handle and repair damage from toxins during this stage of life [17]. Future risk assessment for pesticides may need to take into account a possible increased vulnerability for older adulthood exposures as well as considering the accumulation of effects from even low-level exposures over a person’s lifetime.

### 3.3. PEG and CGEP Studies Chemical Class and Target Organism Class of Indoor and Outdoor Pesticides

**Figure 3.**
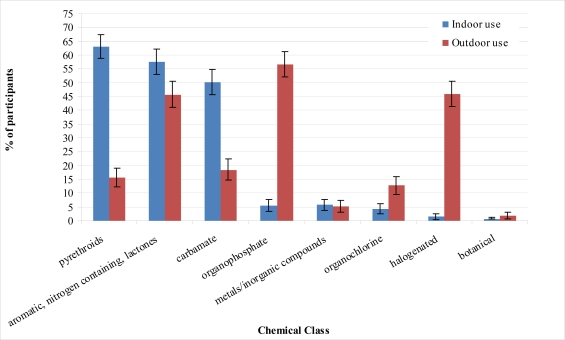
Chemical classes used by PEG and CGEP participants throughout lifetime, chemical class derived from California Department of Pesticide Registry online database and Pesticide Action Network Pesticide Database based on product names reported by participants. (Outdoor use N = 438, Indoor use N = 476).

According to reports in our PEG and CGEP studies, organophosphates, halogenated pesticides, botanicals, and organochlorines were used more often outdoors, while pyrethroids, carbamates, and aromatic, nitrogen containing lactones (e.g., ivermectin, avermectin) were the most common active ingredients in pesticides used indoors. Metal/inorganic pesticides were used both in- and outdoors in similar proportion (Figure 3). However it is important to acknowledge that chemical classes found in consumer use pesticides have changed over the years due to the introduction of new chemicals and regulations limiting use of certain chemicals. For example pyrethroids have only become more common in the past twenty-five to thirty years, while regulations restricted the use of many organochlorines especially for residential and indoor uses [22]. Therefore many individuals in our sample would likely not have used pyrethroids as young adults but also would have decreased their residential use of organochlorines and organophosphates as older adults.

With regard to lifetime frequency of use, the most commonly used chemical classes of pesticides indoors and outdoors were pyrethroids and organophosphates and use increased with age, reflecting the pattern for pesticide use in general (Table 4).

Target organism differed, as expected, depending on where the pesticide was used. Participants who used pesticides outdoors most commonly used herbicides and insecticides (64% and 57% respectively); other outdoor pesticides included nematicides (5%) and disinfectants (<1%). Participants who used indoor pesticides almost exclusively reported using insecticides (93%). Other indoor pesticide use included fungicides (6%), rodenticides and disinfectants (both <1%).

### 3.4. PEG and SUPERB Study Methods of Application

Both indoors and outdoors, pesticides were most commonly applied as sprays when participants were asked to report method ever used (PEG) or method used in the last year (SUPERB). Ever usage of bait and granule varies greatly between indoor and outdoor pesticides; however use of bait and granule is more similar for indoor and outdoor use if we consider their use only in the last year. Participants also reported other application methods for indoor pests, e.g., foggers, gel, and stakes, and for outdoor application, e.g., powder, candles, foam, strips, and traps (Table 5). Some of the differences in methods used over lifetime versus used in the past year may be due to the fact that PEG and CGEP collected data on any type of pesticide over lifetime, while in SUPERB use in the last year focused specifically on insecticide use.

Relatively few participants reported the use of foggers in all three studies. In the case of PEG and CGEP it is possible that participants may have reported foggers as sprays. In SUPERB foggers were clearly separated from sprays. Foggers are a major source of potential insecticide exposure to residential users as they release far more chemical into the environment than the other methods of application mentioned here [23].

### 3.5. Past Year Pesticide Use in SUPERB Study

In total 89% of SUPERB participants reported involvement in at least one of four types of insecticide applications in the past year: indoor application, outdoor application, professional services, or pet products. Insecticides were applied for outdoor pests by 62% and for indoor pests by 35% of the participants, while 37% and 49% of participants reported use of pet products and professional pesticide services, respectively. SUPERB investigated combinations of application types in the past year and found that the majority of participants employed a single (28%) or no more than two application types (34%) (Table 6). Our results agree with a telephone interview study conducted by Flint *et al*. [24] for the California Department of Pesticide Regulation from 2002–2003, in which 51–60% of California participants reported that they, or someone else in their household, had used outdoor pesticides in the 6 months prior to interview. The same study also provided information on pesticide frequency of use, and similar to our results, the majority of participants reported low to moderate use frequencies (<3 times per year).

### 3.6. SUPERB Study Indoor Pesticide Use: Rooms and Areas Treated

Among SUPERB participants, 23% reported using sprays inside their homes in the last year. Of these, 66% applied insecticides to only one room in their home, the remaining applied insecticides to 2 (26%) and 3 rooms (9%). The most commonly treated room was the kitchen (66%). Application in the kitchen creates a possibility for further exposure to insecticides through food that may become contaminated through contact with treated surfaces or surfaces accidentally contaminated with pesticide [25]. Indoor insecticides used in the kitchen were most often applied on surfaces less than one square foot (35%) and in several specific areas ranging in size between one and five square feet (30%), only 22% of participants applied product in cracks and crevices. Similar results were seen in Wu *et al*.’s [16] analysis of SUPERB data for Northern California homes with young children. Keenan *et al*. [23] found that crack and crevice treatments are most effective at limiting pesticide exposure while still effectively eliminating pests. In addition, many other experimental studies utilize crack and crevice application to evaluate pesticide exposure from the pesticide’s dispersal in the environment [26–29]. Data from both SUPERB groups (older adults and families with young children) indicate that crack and crevice application may not be the most commonly used method; therefore, future experimental research to evaluate exposure should consider other application types in order to provide more accurate information for input into exposure models.

### 3.7. SUPERB Study Ventilation and Cleaning After Application of Indoor Pesticides

Data on cleaning after and ventilation during and after application of indoor sprays was only collected from individuals who reported using indoor sprays in the past year (N = 35). Of these individuals, 44% reported opening one or more windows during or after they sprayed insecticides in the room. Sprayed chemicals have been shown to remain at higher concentrations in the air in treated rooms and may disperse into adjacent rooms [27–29]. Ventilation after treatment may reduce the concentration of chemical in the treated room and reduce dispersal [26], yet, our findings suggest that less than half of all individuals using indoor sprays are ventilating rooms during and after applying the sprays. These results are slightly different than the findings from the Northern California SUPERB survey of families with young children, in which 58% reported opening one or more windows during or after application of indoor sprays [16]. This difference could be due to age related factors, or could also be due to the difference in climate between Northern and Central California group. Further investigation will be needed to determine why these differences exist.

Days elapsed between application and cleaning ranged from zero days (22%) to five days (39%). When asked about how rooms were cleaned, 59% reported cleaning only the centers of hardwood floors, 10% reported vacuuming only the centers of carpeted floors, and 71% reported cleaning most of the counters after indoor spraying. Compared to the SUPERB data for Northern California families with young children, older adults allow more days to elapse between application and cleaning, 39% of families with young children cleaned on the day of application, and only 19% waited up to 5 days before cleaning [16]. However areas cleaned (*i.e*., center of floor, most counters) did not differ between older adults and families with young children [16]. It has been shown that pyrethroids, found in our study to be commonly applied indoors, can persist for months on household surfaces unless these surfaces are cleaned [26,29]. If surfaces are not cleaned soon after application, or at all, people may increase their chances of exposure to insecticides through direct and/or indirect contact with treated surfaces and those contaminated accidentally. The disparity in days before cleaning between older adults and families with young children may be due to parents of young children being more concerned about a child frequently coming into contact with pesticide treated surfaces. Therefore parents may attempt to reduce children’s exposure to the insecticides by cleaning soon after application. In homes of older adults where young children may not be frequently present, less urgency may be felt for removing pesticides from treated surfaces through cleaning. However, future study will be needed to definitively determine the reason for differences in post insecticide application behaviors between older adults and families with young children.

### 3.8. SUPERB Outdoor Pesticide Use: Areas Treated

**Figure 4.**
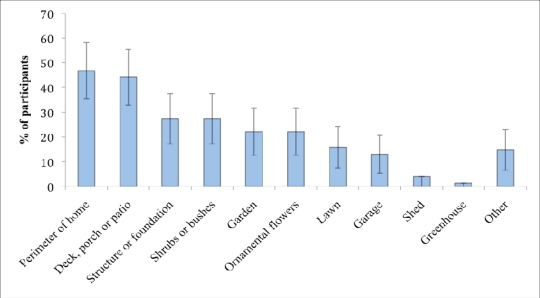
Areas where outdoor sprays were applied in the last year by SUPERB participants (N = 79 *). * Response for bushes, lawn, and other were N = 78, N = 78, and N = 77 respectively.

Participants sprayed anywhere from one to seven areas outside of their homes, but most participants sprayed in only one (36%) or two (30%) areas outdoors. Spraying occurred most often around the perimeter of the home (47%) and on a deck, porch, or patio (44%) (Figure 4). Flint *et al*. [24] reported similar outdoor pesticides application patterns, *i.e*., a majority of participants applied outdoor pesticides to hard outdoor surfaces including building perimeters, base of building, patios, sidewalks, and driveways. Residents may spend substantial amounts of time in these areas, therefore increasing their likelihood of exposure to pesticide residues.

Most participants used outdoor sprays for spot application rather than applying sprays over a large area. While SUPERB asked participants about the general size of the area where insecticides were used, no data was collected in any of the three studies on the amounts of pesticides used during a typical application as participants are unlikely to be able to estimate the mass applied.

### 3.9. PEG and SUPERB Study Personal Protective Equipment Usage During Pesticide Application

Among those PEG participants who had ever applied pesticides outdoors, less than half reported ever using personal protective equipment (PPE), 32% used gloves, 8% used a mask, 2% wore coveralls, and 8% used another type of protection including protective eyewear or combinations of protective wear, such as mask and gloves. Even fewer participants who had applied indoor pesticides at any point during their lifetime reported ever using protection during application. Only 9% used gloves, 2% wore a mask, and 4% used another form of PPE when applying pesticides indoors, e.g., they reported “holding a handkerchief over the mouth” or used several types of PPE in combination. When asked about consistency of PPE use over their lifetime, 16% reported to have always used PPE during outdoor and 5% during indoor pesticide applications. There was no significant difference in PPE use by gender.

We also considered that PPE use could be related to knowledge of, and access to, PPE, therefore we compared PPE use in individuals who had ever farmed to those who had never farmed, however we found no statistically significant difference in PPE use between these two groups. An individual’s pesticide exposure may be reduced by the use of PPE, however our data strongly suggest that most people are not engaging in such exposure reducing behavior, even those who are expected to have access to and more knowledge about the proper use of PPE occupationally. This suggests that individuals perceive residential pesticide application as low risk. Furthermore, many residential pesticides do not instruct the consumer to utilize PPE, which may in part, be a reason for low frequency of use of PPE. In any event, our results indicate that older adults do not engage in any substantial exposure reducing behavior.

In SUPERB, 41% reported using PPE when applying pesticides in the last year. However of the SUPERB participants who reported applying pesticides in the last year, one-third were missing a response to the question regarding PPE use, therefore this percentage may not accurately reflect SUPERB participants’ actual PPE use.

### 3.10. Storage of Pesticides

PEG provided data on storage location. Pesticides applied indoors were most often stored inside the home (46%), while outdoor use pesticides were mainly stored in the garage (60%). For both indoor and outdoor use pesticides additional storage locations included patios or porches and some participants reported discarding the pesticide after the application. SUPERB provided data on storage of all pesticides (not separately for indoor or outdoor products), which were stored most commonly in the garage (50%) followed by inside the home (32%).

We speculate that the storage of pesticides inside the home may be an indicator of, or even lead to, more frequent use by the consumer. If pesticides are stored indoors, they are more readily available, upon notice of pests in a living area, than if they were stored in a garage or outdoors. Additionally, storage inside the home may increase exposure if the chemical is able to contaminate other objects during storage (e.g., by leakage), or the container allows release of vapors into the air.

### 3.11. Limitations

All three surveys used in our studies relied on participants remembering pesticide use occurring anywhere from several months to several decades in the past; therefore our information is limited by the respondents’ ability to recall their pesticide usage over, potentially, many years. Recall error may increase the further back in time the participants were asked to remember. This will contribute to misclassification of exposures, in regards to product types, frequency of exposure and/or the behaviors that contribute to increasing or decreasing exposure, such as PPE, method of application used, and areas treated.

For PEG and CGEP surveys in which participants reported pesticide products over their lifetime without the help of pictures or product lists the identification of product chemical and target classes may have been even more inaccurate and only reflect the most common use patterns. For PEG and CGEP our data collection methods allowed participants to recall as many pesticides as possible, however in our data participants only reported up to five products for each type of pesticide application (*i.e*., indoor or outdoor pesticide use). It is possible that participants used more than five chemicals, and were only able to recall those used most frequently during their lifetime, therefore we may not have captured all pesticides used by participants. The CDPR did not always contain complete information on products of interest, further limiting our ability to identify products and pesticide classes accurately.

## 4. Conclusions

Utilizing data collected from the PEG, CGEP, and SUPERB population-based studies, we have shown that a substantial proportion of older adults in Central California expose themselves to pesticides throughout their lifetimes (albeit at low frequency) with highest use in mid-life. We have shown that use of residential pesticides at younger ages may predict use at older ages, however further study is needed to assess how socioeconomic and lifestyle factors may influence behavioral consistency with regard to pesticide use.

This population predominantly relies on spray applications both during indoor and outdoor use. Older adults are commonly utilizing sprays in the kitchen, which may create opportunities for direct and indirect exposures when pesticides contaminate kitchen surfaces especially since participants also did not report to clean or ventilate areas regularly during and after indoor application. Older adults also apply pesticides in outdoor areas where people may spend time, thereby increasing likelihood of exposures to pesticide residues after application.

Our data also suggests that these populations are not engaging in behaviors to limit their exposure during applications and do not utilize personal protective equipment when applying pesticides.

We have provided a more recent and detailed picture of pesticide use patterns and exposure related behaviors than what has been available in the literature to date for older adults residing in an area of intense agricultural activity. This information will provide more detailed input when creating scenarios for exposure models of current and lifetime pesticide exposure for our unique population and perhaps other comparable populations. These data may also inform future studies assessing the relationships between pesticide exposures and diseases that manifest in older adulthood.

## Figures and Tables

**Table 1.** Comparison of types of data collected in each study (PEG, CGEP, SUPERB) and used to describe residential pesticide usage in older adults residing in Central California.

	**Study**		
**Type of Data Collected**	**PEG**	**CGEP**	**SUPERB**
Lifetime use of pesticides indoors	X	X	X
Type of pesticide applied (target organism)	X	X	X
Lifetime use of pesticides outdoors	X	X	X
Type of pesticide applied (target organism)	X	X	X
Use of insecticides indoors in the last year			X
Room in home			X
Size of area applied			X
Cleaning of area during/after application			X
Ventilation of area during/after application			X
Use of insecticides outdoors in the last year			X
Area where applied			X
Use of personal protective equipment	X		X
Storage of pesticide products	X		X
Method of application	X		X

**Table 2.** Demographics of PEG (N = 359), CGEP (N = 297), and SUPERB (N = 153) studies.

	**Characteristics**	**PEG Total**		**CGEP Total**		**SUPERB Total**	
		**n**	**(%)**	**n**	**(%)**	**n**	**(%)**
Total		359	100.0	297	100.0	153	100.0
Gender							
	Male	185	51.5	116	39.1	52	33.9
	Female	174	48.5	181	60.9	101	66.0
Age							
	50–59	71	19.8	65	21.9	45	29.6
	60–69	98	27.3	120	40.4	58	38.1
	70–79	136	37.9	74	24.9	39	25.6
	80 and over	54	15.0	38	12.8	10	6.6
	Missing					1	
Race							
	White	289	80.5	176	59.3	124	81.0
	Latino	32	8.9	75	25.2	17	11.1
	Black/African American	14	3.9	11	3.7	2	1.3
	Asian/Pacific Islander	10	2.8	9	3.0	1	0.6
	Other	14	3.9	22	7.4	7	4.5
	Don’t Know/Refused			4	1.3	2	1.2
Education							
	<12 years (did not graduate high school)	33	9.2	46	15.6	7	4.6
	12 years (High school graduate)	129	35.9	65	22.1	56	36.8
	>12 year	197	54.9	183	62.2	89	58.6
	Missing			3		1	

**Table 3.** (**a**) Correlation of pesticide use (yes/no) outdoors in younger adulthood and older adulthood from SUPERB, PEG, and CGEP studies (N = 809); (**b**) Correlations of pesticide use (yes/no) indoors in younger and older adulthood from SUPERB, PEG, and CGEP studies (N = 809).

**(a)**				
	
	**Pesticide Use Outdoors During Younger Adulthood (ages <45)**	**Pesticide Use Outdoors During Older Adulthood (ages ≥45)**		
	
		**No**	**Yes**	**Total**
	
	**No**	232 (29%)	174 (21%)	406 (50%)
	**Yes**	70 (9%)	333 (41%)	403 (50%)
	
	**Total**	302 (38%)	507 (62%)	809 (100%)
	rφ = 0.41, p-value < 0.0001.			

**(b)**				
	
	**Pesticide Use Indoors During Younger Adulthood (ages <45)**	**Pesticide Use Indoors During Older Adulthood (ages ≥45)**		
	
		**No**	**Yes**	**Total**
	
	**No**	214 (26%)	144 (18%)	358 (44%)
	**Yes**	104 (13%)	347 (43%)	451 (56%)
	
	**Total**	318 (39%)	491 (61%)	809 (100%)
	rφ = 0.37, p-value < 0.0001.			

**Table 4.** Frequency of outdoor organophosphate and indoor pyrethroid use over lifetime by PEG and CGEP participants.

Organophosphates (outdoor use) N = 248 *									Pyrethroid (indoor use) N = 301 *							

	**Young Adult (<25 years)**		**Adult (25–44 years)**		**Middle Age (45–64 years)**		**Senior^1^(≥65 years)**		**Young Adult (<25 years)**		**Adult (25–44 years)**		**Middle Age (45–64 years)**		**Senior^1^(≥65 years)**	
**Frequency of Use**	**Freq**	**(%)**	**Freq**	**(%)**	**Freq**	**(%)**	**Freq**	**(%)**	**Freq**	**(%)**	**Freq**	**(%)**	**Freq**	**(%)**	**Freq**	**(%)**
Low use (≤1 time/year)	237	95.6	195	78.9	168	67.7	148	77.5	248	83.5	183	61.2	168	55.8	95	56.5
Moderate use (2–11 times/year)	10	4	43	17.4	72	29	42	22	34	11.4	91	30.4	107	35.5	61	36.3
High use (1 time/week to 1 time/month)	1	0.4	9	3.6	8	3.2	1	0.5	15	5	25	8.4	26	8.6	12	7.1
*Total*	*248*	*100*	*247*	*100*	*248*	*100*	*191*	*100*	*297*	*100*	*299*	*100*	*301*	*100*	*168*	*100*

* Missing values in age groups <25year, 25–44 year, 45–64 years are due to not all participants responding for all age groups or responding “don’t recall”;

1 Missing values in age group ≥65 are in part due to not all participants having reached the age of 65 at the time of interview.

**Table 5.** Method of outdoor and indoor pesticide application reported by PEG (ever used method) and SUPERB (used method in the last year).

	**Lifetime Use of Application Methods (PEG N = 356)**			**Use of application Methods within the last year (SUPERB N = 153)**		
	
**Application Methods Used for Pests**	Frequency *	Percent * (%)	**95th% Confidence interval (%)**	Frequency *	Percent * (%)	**95th% Confidence interval (%)**
*Outdoor*	*256*	*71.9*	*[67.2, 76.6]*	*95*	*62.1*	*[54.3, 69.9]*
spray	214	83.6	[79.0, 88.2]	77	81.0	[73.0, 89.1
bait	52	20.3	[15.3, 25.3]	31	32.6	[23.0, 42.2]
granule	55	21.5	[16.4, 26.5]	17	17.9	[10.0, 25.7]
Other **	45	17.6	[12.9, 22.3]	15	15.8	[8.3, 23.3]
*Indoor*	*269*	*75.1*	*[70.6, 79.6]*	*54*	*35.3*	*[27.6, 42.9]*
spray	233	86.6	[82.5, 90.7]	35	64.8	[51.7, 78.0]
bait	20	7.4	[4.3, 10.6]	17	31.5	[18.7, 44.3]
granule	10	3.7	[1.4, 6.0]	11	20.4	[9.3, 31.5]
Other ^^^	71	26.4	[21.1, 31.7]	9	16.7	[6.4, 26.9]
fogger ^º^	…	…	…	8	5.2	[1.7, 8.8]

* Frequency does not add to number of subjects, nor does total percentage equal 100% because participants could choose multiple methods of application;

** Outdoor other category includes: powder, candles, foam, strips, traps, or liquid;

^ Indoor other category include: powder, candles, foam, fogger, strips, traps, stakes, gel;

º Only SUPERB specifically questioned participants about use of foggers.

**Table 6.** Types of pesticide applications used in combination within the last year by SUPERB participants (N = 153).

**Insecticide applications used in combination**	**Freq.**	**(%)**	**95th% Confidence Interval (%)**
*No insecticide use reported*	*17*	*11.1*	*[6.1, 16.1]*

*Single product used*	*43*	*28.1*	*[20.9, 35.3]*
Outdoor only	15	34.8	
Indoor only	3	6.9	
Pet only	9	20.9	
Professional only	16	37.2	

*Combination of 2 application types*	*52*	*34.0*	*[26.4, 41.6]*
Outdoor and indoor	15	28.8	
Outdoor and professional	17	32.7	
Other	20	38.5	

*Combination of 3 application types*	*30*	*19.6*	*[13.2, 25.8]*
Outdoor, indoor, and pet	10	33.3	
Outdoor, indoor, and professional	10	33.3	
Outdoor, pet, and professional	10	33.3	

*Combination of 4 application types (all)*	*11*	*7.2*	*[3.0, 11.3]*

*Total*	*153*	*100.0*	
